# Downregulation of thioredoxin-like 1 contributes to miconazole’s antiviral activity against West Nile virus

**DOI:** 10.3389/fcimb.2026.1789670

**Published:** 2026-07-21

**Authors:** Liancheng Xia, Zhenghan Luo, Yanhua He, Haoran Peng, Xu Zheng, Xijing Qian, Cuiling Ding, Ping Zhao, Yangang Liu, Zhongtian Qi

**Affiliations:** 1 Department of Microbiology, Faculty of Naval Medicine, Naval Medical University, Shanghai, China; 2 Key Laboratory of Biological Defense, Ministry of Education, Naval Medical University, Shanghai, China; 3 Department of Infectious Disease Prevention and Control, Center for Disease Prevention and Control of Eastern Theater of the Chinese People's Liberation Army, Nanjing, Jiangsu, China

**Keywords:** antiviral drug, drug repurposing, miconazole, thioredoxin-like 1, viral replication, West Nile virus

## Abstract

**Introduction:**

West Nile virus (WNV)-associated neurological diseases pose a global public health burden, yet no approved antiviral treatments are available. This is primarily due to the challenges of crossing the blood-brain barrier and the lengthy, costly process of drug development.

**Methods:**

High-throughput screening of a blood-brain barrier-penetrating FDA-approved compound library was performed to identify anti-WNV candidates in a neuronal cell infection model. Antiviral efficacy was further evaluated across different target cells and a range of flaviviruses, as well as in a mouse model of central nervous system (CNS) WNV infection. Stage-of-action analysis and mechanistic studies were conducted.

**Results:**

Miconazole emerged as a promising candidate with significant antiviral efficacy against multiple flaviviruses in different cell types. In the CNS infection mouse model, miconazole treatment reduced viral load in brain tissue and improved survival rates. The compound primarily inhibited viral replication, without affecting binding, internalization, or membrane fusion. Mechanistic studies suggested that this antiviral effect may be mediated through the downregulation of host protein thioredoxin-like 1 (TXNL1).

**Discussion:**

Our findings highlight miconazole as a promising candidate for repurposing in anti-flavivirus therapy and identify TXNL1 as a potential host target for the development of broad-spectrum antivirals.

## 1 Introduction

West Nile virus (WNV) is an enveloped, positive-sense single-stranded RNA virus primarily transmitted by Culex mosquitoes (Andreadis, 2012). It belongs to the Flaviviridae family, which also includes yellow fever virus (YFV), Japanese encephalitis virus (JEV), tick-borne encephalitis virus (TBEV), dengue virus (DENV), and Zika virus (ZIKV) (Petersen et al., 2003). WNV was first isolated from a patient in Uganda in 1937 and was introduced to North America in 1999, causing a large outbreak of meningoencephalitis (Habarugira et al., 2020; Petersen et al., 2003). Today, it ranks among the leading causes of mosquito-borne encephalitis worldwide, with over 60,000 neuroinvasive cases and 3,100 deaths (CDC ArboNET West Nile Virus Historical Data, 2025; Hadfield et al., 2019).

Similar to other neuroinvasive flaviviruses such as JEV and TBEV, WNV can cross the blood–brain barrier (BBB) and enters the central nervous system (CNS) (Liu et al., 2020). Most infections are mild, but about 1% of cases progress to severe neuroinvasive conditions such as encephalitis, meningitis or severe flaccid paralysis with high morbidity and mortality (Luque-Ambrosiani et al., 2025). Therefore, BBB permeability is a critical requirement for drugs targeting CNS infections. In fact, the highly selective nature of the BBB, which protects the CNS from pathogens and toxins, also poses a major obstacle for drug delivery, as over 98% of small-molecule drugs and nearly all large-molecule therapeutics are unable to cross it under normal conditions. To overcome this challenge, various strategies have been explored, including the use of receptor-mediated transcytosis, nanoparticle-based carriers, focused ultrasound, or viral vectors, all aimed at enhancing CNS drug penetration without compromising BBB integrity. In the context of WNV and related neuroinvasive flaviviruses, improving BBB permeability of antiviral agents could significantly reduce the risk of severe neuroinvasive outcomes such as encephalitis or flaccid paralysis.

Although numerous compounds have shown anti-WNV activity in cell culture or murine models (Qian and Qi, 2022; Yao et al., 2019; Zheng et al., 2024), few have entered clinical trials, and no drug has been approved for human use (Sinigaglia et al., 2020). This gap arises from two major challenges: first, the complex and resource-intensive nature of drug development leads to prolonged timelines and substantial costs (Wu et al., 2025); second, many reported anti-WNV candidates may lack sufficient BBB permeability (Zheng et al., 2024). For example, compounds such as epigallocatechin-3-gallate, nordihydroguairetic acid, and etravirine were previously reported to exhibit significant anti-WNV activity (Merino-Ramos et al., 2017; Wang C. Y. et al., 2018; Zheng et al., 2024), but their relatively low BBB permeability may limit their therapeutic efficacy in clinical settings.

Drug repurposing offers a valuable strategy to accelerate the development of antiviral therapies, leveraging existing safety profiles to shorten timelines and reduce costs (Ferraz et al., 2020; Hamid et al., 2024; Harisha et al., 2025). Moreover, this approach can deepen our understanding of drug targets and the molecular mechanisms of viral infection (Martin and Cheng, 2020; Imran et al., 2025). In this study, we performed phenotypic screening of an FDA-approved, BBB-permeable drug library to identify anti-West Nile virus (WNV) candidates. This CNS−accessible library complements target−based screening and can uncover new host or viral targets while providing mechanistic insights. Using this strategy, we identified some anti−WNV compounds, including miconazole and rimonabant. Miconazole exhibited broad−spectrum anti−flaviviral activity *in vitro* and protected against WNV−induced neuronal infection and lethality in a mouse model of WNV encephalitis. Subsequent mechanistic studies elucidated the molecular basis of miconazole’s antiviral action, further advancing our understanding of WNV replication and suggesting a potential therapeutic option for WNV infection.

## 2 Results

### 2.1 High-throughput screening for anti-WNV compounds

**Figure 1:**
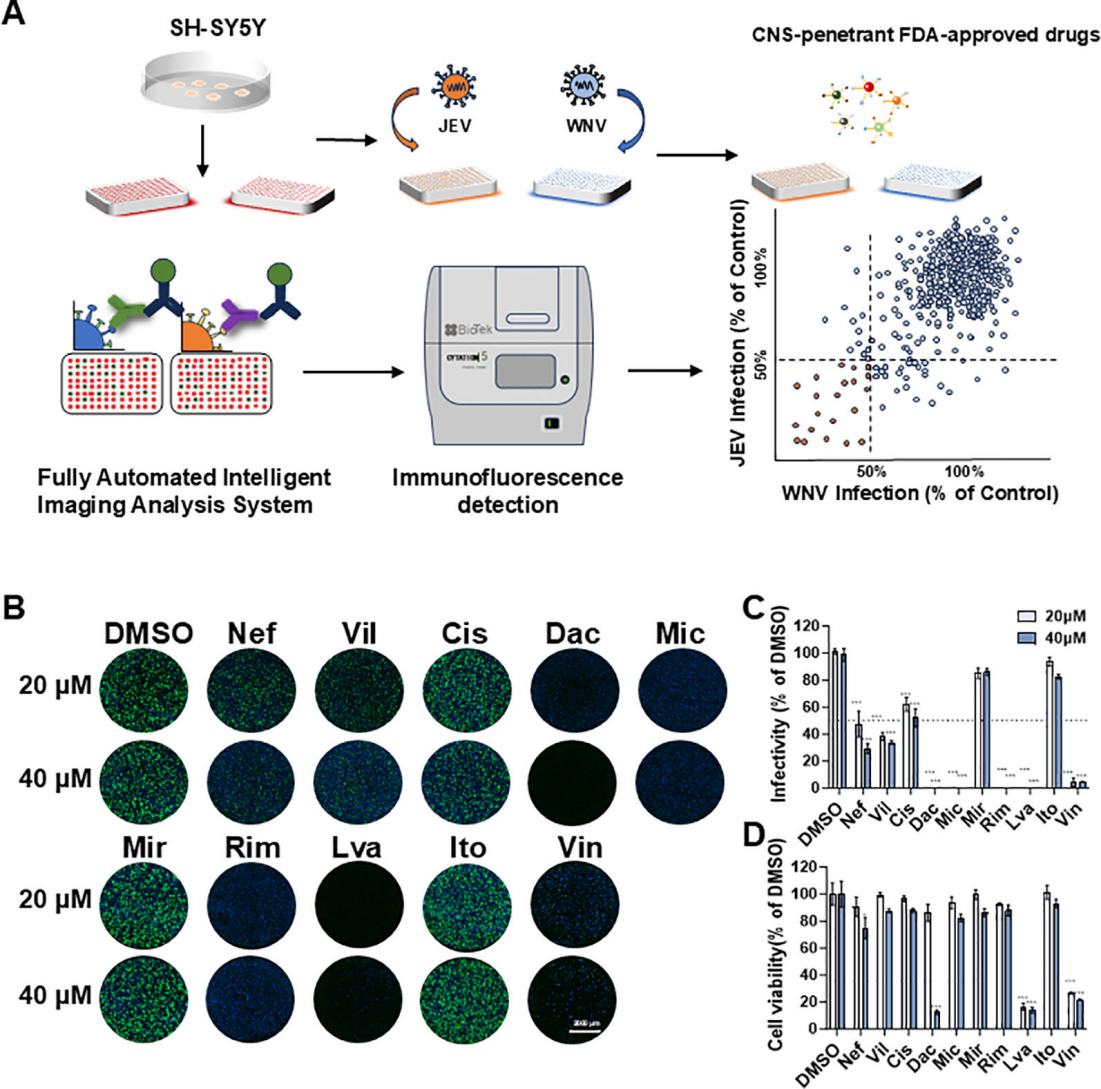
Screening of anti-WNV candidates from a BBB-penetrant FDA-approved drug library. **(A)** Schematic of high-throughput screening against WNV and JEV. **(B, C)** SH-SY5Y cells infected with WNV (MOI = 0.3) in the presence of 10 candidate compounds (20 and 40 μM) for 4 hours. **(B)** Representative immunofluorescence images (virus antigen, green; DAPI, blue). **(C)** Quantification of infected cells normalized to DMSO control. **(D)** Cell viability assessed by CCK-8 assay. Abbreviations: Mic, miconazole nitrate; Nef, neflamapimod; Vil, vilazodone hydrochloride; Dac, dacomitinib; Cis, cisatracurium besylate; Mir, mirtazapine; Rim, rimonabant; Lva, lvacaftor; Ito, itopride hydrochloride; Vin, vinorelbine ditartrate. Data represent the mean ± SD of three biologically independent experiments (n = 3). *P < 0.05, ***P < 0.001 vs. DMSO control.

To develop drugs that alleviate neuropathology caused by mosquito-borne flaviviruses, we conducted a phenotypic screen using an FDA-approved library of blood-brain barrier (BBB)-penetrant small molecules. We first prioritized drugs with BBB permeability from the FDA-approved drug library to establish a dedicated compound sub-library (722 compounds). The screening workflow is illustrated in **Figure 1A**: SH-SY5Y cells infected with WNV (MOI = 0.3) or JEV (MOI = 0.3) were treated with the sub-library (20 µM), and viral infectivity was determined by immunofluorescence (IF) at 24 hours post-infection (hpi). Drugs exhibiting >50% inhibition against both viruses and with no prior confirmed antiviral activity were selected for second-round validation. In the second-round validation, virus-infected cells were treated with candidate drugs at two concentrations (20 and 40 μM), and cytotoxicity was evaluated using the CCK-8 assay kit. The results demonstrated that neflamapimod, vilazodone hydrochloride, rimonabant, and miconazole markedly inhibited viral infection, whereas dacomitinib, ivacaftor, and vinorelbine ditartrate significantly reduced cell viability (**Figures 1B–D**). Notably, miconazole at 20 μM exhibited approximately 100% viral inhibition with minimal impact on cell viability, suggesting that it represents a promising candidate antiviral agent.

### 2.2 Miconazole exhibits broad-spectrum anti-flavivirus activity

**Figure 2:**
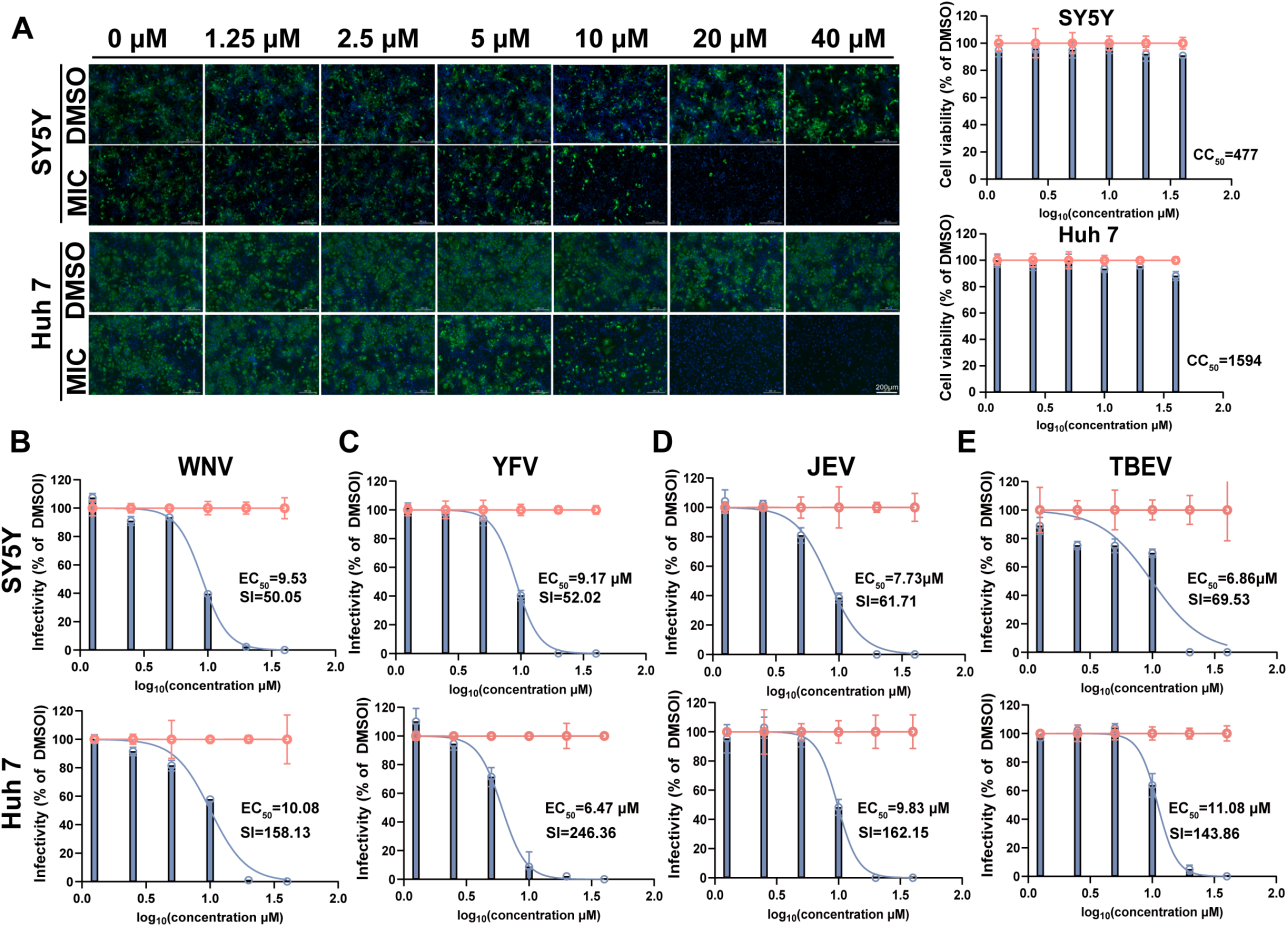
Broad-spectrum antiviral activity of miconazole against flaviviruses. **(A)** SH-SY5Y and Huh7 cells were infected with WNV (MOI = 0.3) and treated with serial dilutions of miconazole (0–40 μM) for 24 h. Left: representative immunofluorescence images; right: cell viability (CCK-8). EC_50_ and CC_50_ values were determined by nonlinear regression. **(B)** Quantification of WNV-infected cells from **(A, C–E)** Huh7 and SH-SY5Y cells infected with YFV, JEV, or TBEV (MOI = 0.3) were treated with miconazole for 24 hours. Viral infectivity was determined by immunofluorescence. Data represent the mean ± SD of three biologically independent experiments (n = 3).

Miconazole, an FDA-approved antifungal medication, has shown potential against SARS-CoV-2 in an in-silico repurposing study [19]. However, no report has confirmed its antiviral activity, and research on cultured Aedes albopictus (C6/36) mosquito cells indicated that miconazole might even stimulate DENV replication [20]. To assess miconazole’s anti-WNV activity in different cell types, we examined its dose response in human cell lines Huh7 and SH-SY5Y. Following WNV infection, cells were treated with six 2-fold serial dilutions of miconazole, ranging from 40 µM to 1.25 µM (excluding the 0 µM control). At 24 hpi, the proportion of WNV-infected cells was measured using immunofluorescence. Concurrently, cell viability was assessed using a CCK-8 kit to evaluate the cytotoxicity of various miconazole concentrations. As shown in the left panels of **Figures 2A, B**, WNV infectivity in both cell types decreased as miconazole concentrations increased. The selectivity index (SI) values were 158.13 for Huh7 cells and 50.05 for SH-SY5Y cells, with no significant toxicity observed at any dose tested (right panels of **Figures 2A, B**).

To further investigate the impact of miconazole on other flavivirus family members, we examined its effects on YFV, JEV, and TBEV—the latter two being neurotropic flaviviruses. The findings revealed that miconazole exerted a dose-dependent inhibitory effect on YFV, JEV, and TBEV in both cell types. In SH-SY5Y cells, the SI values were 52.02, 61.71, and 69.53, respectively, while in Huh7 cells, the SI values were 246.36, 162.15, and 143.86, respectively (**Figures 2C–E**). These results demonstrate the broad-spectrum antiviral activity of miconazole against flaviviruses in different target cells, suggesting that it may target a common host factor essential for the flavivirus life cycle.

### 2.3 Miconazole protects mice from WNV infection-induced lethality

**Figure 3:**
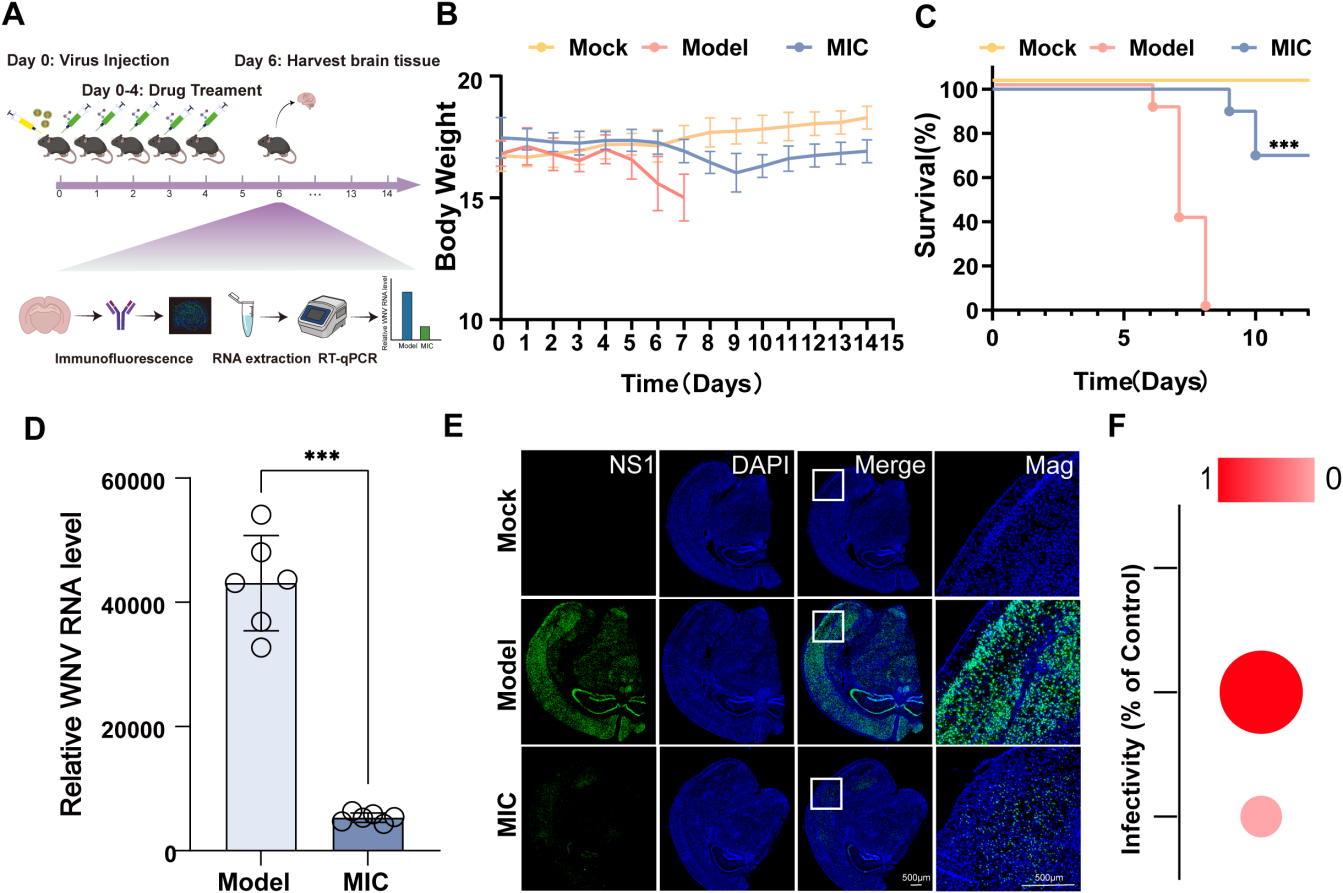
Miconazole protects mice from lethal WNV infection. Mice were intracranially inoculated with WNV (100 PFU) and treated with miconazole (50 mg/kg) or vehicle daily for 5 days (0–4 dpi). **(A)** Experimental timeline. **(B, C)** Body weight and survival (n = 10). **(D)** Brain viral load at 6 dpi by RT-qPCR (n = 3). **(E, F)** Brain sections stained for WNV-NS1 (green) and DAPI (blue); boxed areas magnified in insets. Scale bars, 500 μm. ***P < 0.001 vs. model group.

To evaluate the *in vivo* antiviral efficacy of miconazole, we examined its protective effects in mice challenged with WNV. Mice that received an intracranial injection of a lethal dose of WNV were treated with either miconazole or DMSO in corn oil (solvent control) via daily oral gavage from day 0 to day 4 post-infection (dpi) (**Figure 3A**). Body weight and survival were monitored until 14 dpi. Mice in the model group (infected and treated with DMSO in corn oil) exhibited obvious symptoms such as tremors and limb weakness at 3 dpi, with body weight beginning to decline at 5 dpi (**Figures 3B, C**). Mortality occurred at 6 dpi, and by 8 dpi, all mice in the model group had succumbed. In contrast, miconazole-treated mice showed significantly alleviated symptoms, with delayed and less severe weight loss, reaching a nadir at 9 dpi before recovering, and demonstrated a final survival rate of 66.7% (**Figures 3B, C**). At 6 dpi, brain samples were collected from each group, and viral loads in brain tissue were assessed using RT-qPCR and tissue immunofluorescence. Consistent with the survival analysis, repeated miconazole administration markedly reduced viral load in the brain compared to the model group (**Figure 3D**). Immunofluorescence assays for WNV-NS1 antigen in mouse brains showed that WNV was widely present in brain sections from the model group and was dramatically diminished in miconazole-treated mice (**Figures 3E, F**). These findings demonstrate that miconazole can reduce viral load in the CNS and protect mice from the lethal effects of WNV infection, indicating that miconazole represents a potential therapeutic option against WNV.

### 2.4 Miconazole exerts antiviral effects primarily in the replication stage

**Figure 4:**
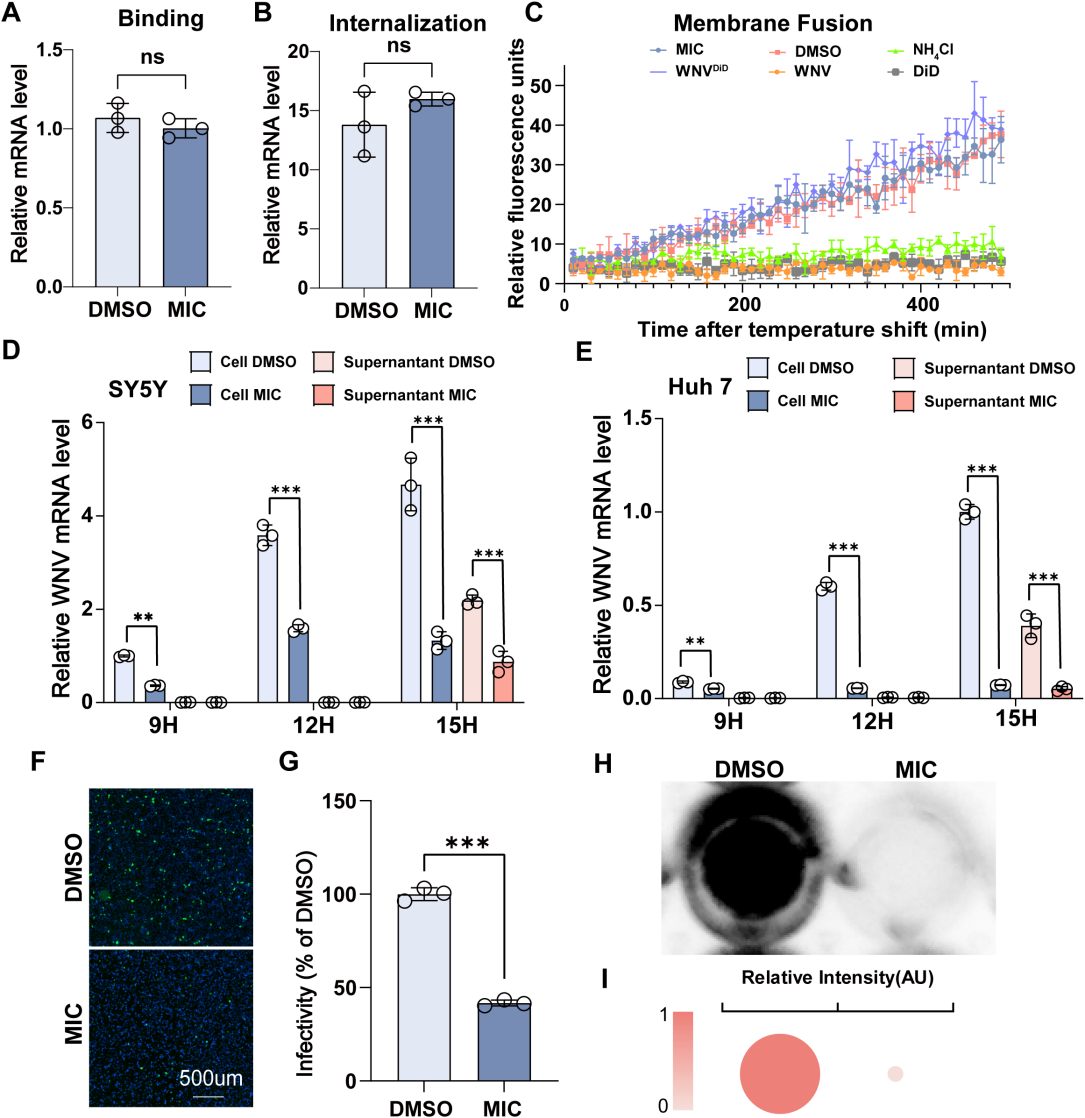
Miconazole inhibits WNV replication. **(A)** Viral binding assay: SH-SY5Y cells incubated with WNV (MOI = 10) and miconazole (20 μM) at 4 °C for 90 min; bound viral RNA quantified by RT-qPCR. **(B)** Viral internalization assay: cells treated as in **(A)**, shifted to 37 °C for 2 h, proteinase K-treated, and analyzed by RT-qPCR. **(C)** Membrane fusion assay: WNV^DiD^ (MOI = 1)-infected cells treated with miconazole (20 μM), NH_4_Cl (25 mM), or DMSO; fluorescence recorded every 15 min. DiD: DiD dye alone; WNV: unlabeled virus. **(D, E)** WNV genome RNA-transfected SH-SY5Y **(D)** and Huh7 **(E)** cells treated with miconazole (20 μM); viral RNA in cells and supernatant quantified at 9, 12, and 15 hpt. **(F–I)** WNV replicon-transfected SH-SY5Y cells treated with miconazole (20 μM) for 48 hours: **(F, G)** NS1 immunofluorescence; **(H, I)** luciferase activity. Data represent the mean ± SD of three biologically independent experiments (n = 3). ns, not significant; **P < 0.01, ***P < 0.001 vs. DMSO control.

To determine the stage of the WNV life cycle targeted by miconazole, we first examined its effects on viral entry, including binding, endocytosis, and membrane fusion, in SH-SY5Y cells. For the binding assay, WNV (MOI = 10) was incubated with cells on ice for 2 h in the presence of miconazole (20 µM) or solvent control. Following washing, WNV RNA levels were quantified by RT-qPCR. As shown in **Figure 4A**, miconazole did not inhibit WNV attachment to the cell surface. For the internalization assay, cells were shifted to 37 °C for 1.5 h after binding, and extracellular virions were removed by proteinase K treatment. Intracellular WNV RNA was then measured by RT-qPCR. The results indicated that miconazole does not impair viral internalization (**Figure 4B**). To assess membrane fusion, we utilized DiD-labeled WNV (WNV^DiD^). DiD incorporated into the viral envelope is initially fluorescence-quenched; fusion with host membranes dilutes the dye, resulting in dequenching and increased fluorescence. As shown in **Figure 4C**, unlike the fusion inhibitor NH4Cl (positive control), miconazole did not significantly alter fluorescence intensity, indicating that it does not inhibit viral-endosomal membrane fusion.

We next investigated the effect of miconazole on viral replication. WNV genomic RNA was transfected into SH-SY5Y and Huh7 cells using Lipofectamine. At 6 h post-transfection (hpt), cells were treated with miconazole (20 µM) or DMSO, and viral RNA levels in cells and supernatants were measured by RT-qPCR at indicated time points. Intracellular viral RNA levels were low at 9 hpt but increased markedly by 12 hpt, with no detectable RNA in the supernatant, indicating active replication prior to release (**Figures 4D, E**). By 15 hpt, viral RNA was detected in both cellular and supernatant fractions, confirming the onset of the release phase. Notably, miconazole potently suppressed intracellular viral RNA at all time points examined, demonstrating robust inhibition of WNV RNA replication. To further validate these findings, we employed a WNV replicon harboring a NanoLuc reporter gene. Following replicon RNA transfection, cells were treated with miconazole or DMSO, and NS1 protein expression and luciferase activity were assessed at 48 hpt. Miconazole significantly reduced WNV NS1 expression and replicon luciferase activity (**Figures 4F–I**), confirming that miconazole targets the replication stage of WNV infection.

### 2.5 Downregulation of thioredoxin-like 1 contributes to miconazole’s antiviral activity

**Figure 5:**
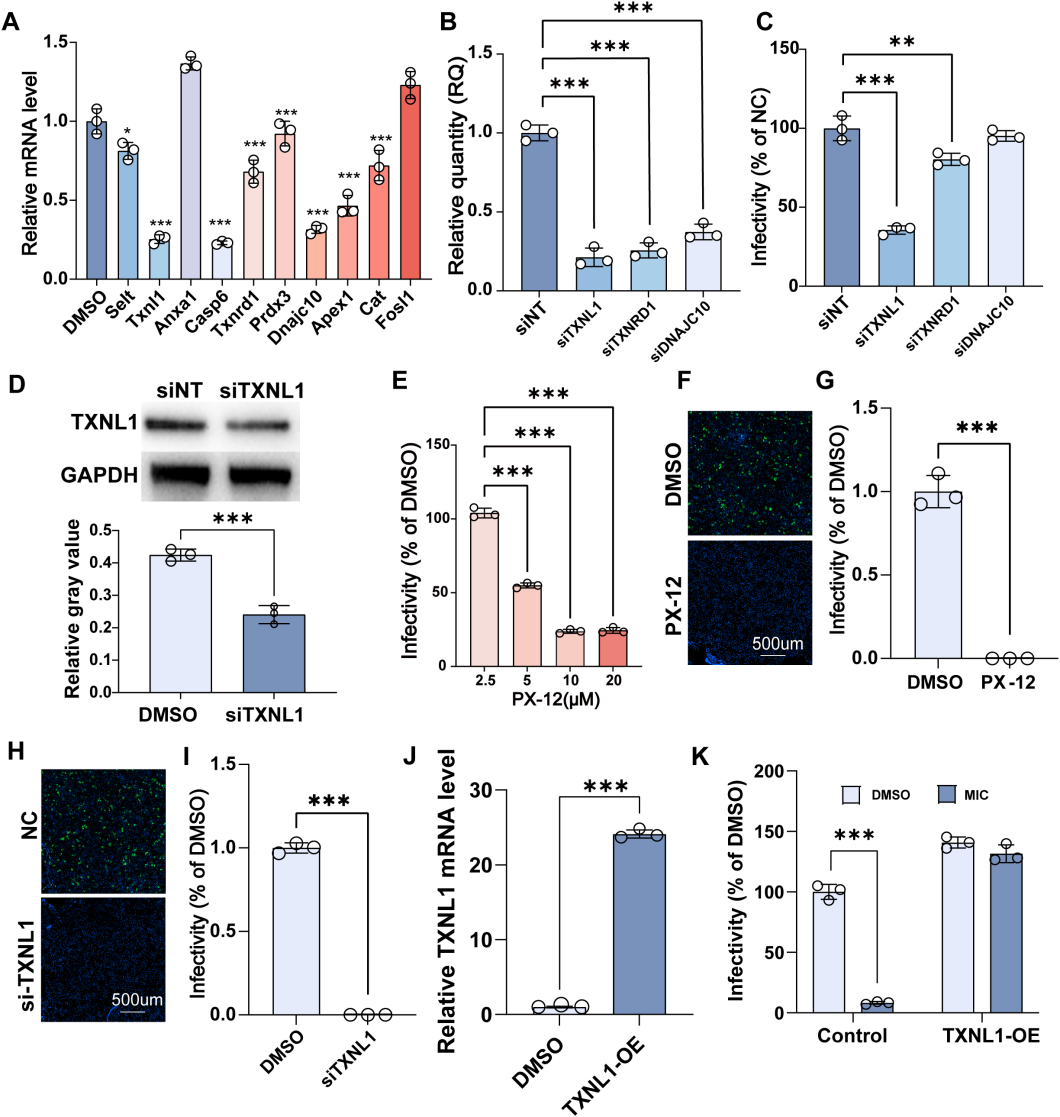
Miconazole inhibits WNV replication via TXNL1 downregulation. **(A)** SH-SY5Y cells were treated with miconazole (20 μM) for 24 hours; candidate gene mRNA levels were measured by RT-qPCR. **(B–D)** Cells were transfected with siRNAs targeting TXNRD1, TXNL1, or DNAJC10 for 48 hours: **(B)** target mRNA levels; **(C)** WNV infection rate (MOI = 0.3, 24 hours); **(D)** TXNL1 protein level by western blot. **(E)** Cells were infected with WNV and treated with the TXNL1 inhibitor PX-12 (0–20 μM). **(F, G)** WNV replicon-transfected cells were treated with PX-12 (20 μM); NS1 expression was analyzed by immunofluorescence; The fluorescence intensity percentage was calculated. **(H, I)** siTXNL1-transfected cells were subsequently transfected with WNV replicon; NS1 expression was analyzed by immunofluorescence; The fluorescence intensity percentage was calculated. **(J)** TXNL1 OE plasmid-transfected cells were subsequently transfected with WNV replicon, relative TXNLF1 mRNA level was determined by qRT-PCR. **(K)** WNV NS1 expression was analyzed by immunofluorescence and the fluorescence intensity percentage was calculated. Data represent the mean ± SD of three biologically independent experiments (n = 3). *P < 0.05, **P < 0.01, ***P < 0.001 vs. DMSO or siNT control.

Previous studies have shown that miconazole exerts antifungal activity by targeting fungal ergosterol biosynthesis (Heeres et al., 2010); however, the mechanism by which it suppresses viral infection remains elusive. Given that miconazole-induced transcriptome changes in neonatal rat cardiomyocytes have been identified by DNA microarray analysis (Won et al., 2012), and considering that viral replication depends on host factors, we hypothesized that miconazole may exert antiviral effects by downregulating specific host factors essential for the viral life cycle. To test this hypothesis, we first examined the expression of host genes reported to be downregulated by miconazole (Won et al., 2012). As shown in **Figure 5A**, among 10 candidate genes, TXNL1, TXNRD1, and DNAJC10 were significantly downregulated by >50% following miconazole treatment. To functionally validate these candidates, siRNA pools targeting each gene were designed. RT-qPCR confirmed efficient knockdown by all three siRNA pools (**Figure 5B**). Notably, TXNL1 silencing resulted in the strongest inhibition of WNV infection (64.48% reduction; **Figure 5C**), with knockdown efficiency verified at the protein level by Western blot (**Figure 5D**). Due to the lack of a specific inhibitor for TXNL1, and given that TXNL1 is a member of the thioredoxin superfamily and shares structural homology with thioredoxin-1 (Trx1), we employed PX-12-a well-characterized irreversible inhibitor of Trx1 that targets its Cys73 residue-as a tool compound to explore the functional involvement of the thioredoxin superfamily in WNV replication. As shown in **Figures 5E**, PX-12 exhibited dose-dependent inhibition of WNV infection. To verify whether TXNL1 participates in the process of viral replication, we then assessed the impact of TXNL1 knockdown by siRNA and PX-12 treatment on WNV replicon expression. As anticipated, both TXNL1 downregulation and PX-12 treatment nearly abolished WNV replication (**Figures 5F–I**). Furthermore, we transfected SY5Y cells with a TXNL1-overexpressing plasmid and examined its effects on viral infection and the antiviral activity of miconazole. As shown in **Figures 5J, K**, TXNL1 overexpression promoted WNV infection and significantly rescued the infection in the presence of miconazole. Collectively, these findings indicate TXNL1 is a likely contributing factor in miconazole’s inhibition of WNV infection.

## 3 Discussion

Despite the lack of approved antiviral therapeutics for WNV infection and WNND, the recent resurgence of outbreaks underscores the urgent need for effective countermeasures (García San Miguel Rodríguez-Alarcón et al., 2021; Hamid et al., 2024). This study employed a high-throughput screening that showed miconazole has broad-spectrum efficacy against various flaviviruses. *In vivo*, miconazole treatment significantly reduced viral load in brain tissue, alleviated weight loss, delayed death, and improved survival rates in WNV-infected mice. Further exploration of the mechanism revealed that miconazole suppresses the viral replication stage, but not binding, internalization, or membrane fusion processes, and its activity was associated with the downregulation of TXNL1 expression.

While miconazole’s role in combating flaviviruses remains unreported, some derivatives was shown to exhibit anti-flavivirus activity. One study identified two azole analogs with significant efficacy in inhibiting dengue virus replication. Additionally, several thiazole derivatives synthesized in a recent study demonstrated notable inhibition of DENV2 NS2b-NS3 protease (McDowell et al., 2010; Murtuja et al., 2025). The imidazole class of antifungal agents has demonstrated varying degrees of antiviral activity against several viruses, including dengue virus, enterovirus, hepatitis C virus (HCV), and human immunodeficiency virus (HIV), suggesting that related compounds may possess untapped antiviral potential (Lee et al., 2000; Kim et al., 2022, Swain and Mohanty, 2019). Nonetheless, a systematic evaluation of miconazole as an antiviral agent is still lacking.

Previous studies on small-molecule inhibitors for flaviviruses have mainly concentrated on *in vitro* antiviral efficacy, often neglecting the crucial factor of blood-brain barrier (BBB) permeability. This oversight has hindered the clinical translation of treatments for neurotropic infections. In our study, we screened a library of FDA-approved drugs known for their BBB permeability. This drug repurposing strategy utilizes established safety profiles to accelerate clinical development and reduce costs. Additionally, the candidate molecules identified through this approach could offer mechanistic insights relevant to other neurotropic viruses via host-directed pathways. Through this strategy, we identified promising anti-WNV candidates, including miconazole and rimonabant, in a neuronal cell infection model. While this study focuses on miconazole, the role of rimonabant will be systematically evaluated in future investigations. Although our screening employed neural-derived target cells, we observed that miconazole exhibits broad-spectrum anti-flaviviral activity extending beyond neuroinvasive viruses, warranting further investigation of its efficacy against non-neurotropic flaviviruses. The consistent antiviral activity across neuronal and peripheral cell types implies a mechanism involving ubiquitously expressed host factors. This hypothesis is supported by our identification of TXNL1—a protein expressed across all tissues at varying levels (Zhao and Qi, 2021)—as a potential target of miconazole’ antiviral effects.

Notably, our findings highlight an important caveat for antiviral drug development: relying solely on efficacy data from peripheral infection models may cause promising candidates against CNS-tropic viruses to be overlooked. This disconnect between conventional screening models and clinical requirements may explain why many antiviral candidates fail in clinical translation in antiviral drug development. Therefore, for viruses with known neurotropism, direct evaluation using CNS infection models provides a more rigorous assessment of antiviral efficacy. We evaluated *in vivo* efficacy using an intracerebral WNV challenge model in wild-type C57BL/6 mice. This model was selected because C57BL/6 mice exhibit high susceptibility to WNV, developing severe neurological disease and succumbing to infection (Beasley et al., 2002; Borisevich et al., 2006). Intracerebral inoculation ensured consistent, high-titer viral replication within brain parenchyma, providing a robust system for assessing miconazole’s antiviral activity in the CNS. Continuous miconazole administration significantly improved survival rates and reduced viral load in brain tissue, supporting its potential as a therapeutic candidate against neuroinvasive flavivirus infections.

According to FDA guidelines, the mouse-to-human equivalent dose of 50 mg/kg/day in mice is approximately 4–6 mg/kg/day based on body surface area. Clinically, miconazole is typically administered intravenously at doses ranging from 200–1000 mg/day (approximately 3–15 mg/kg/day for a 70 kg adult) (Davis and Donovan, 1979). The oral formulation has relatively low bioavailability (about 25–30%), leading to reduced systemic exposure compared to intravenous administration (Song et al., 2019). Despite this, the oral dose used in this study (50 mg/kg/day in mice) achieved significant antiviral efficacy in the brain, suggesting that the effective human equivalent dose may be within the clinically achievable range. Besides, miconazole has a half-life of approximately 20–25 hours in humans. Following an 800 mg intravenous infusion, cerebrospinal fluid (CSF) concentrations of miconazole reach 0.1–0.3 µg/mL within one hour, and the drug has been successfully used to treat fungal meningitis (Sung et al., 1978). In our study, once-daily oral administration effectively inhibited WNV replication in the brain, indicating that the drug can achieve CNS concentrations sufficient to suppress WNV despite its moderate oral bioavailability. For refractory CNS infections, intrathecal administration offers an alternative route. In patients with coccidioidal meningitis, intrathecal injection of 20 mg miconazole produced CSF concentrations of 6.5, 2.4, 0.77, and 0.24 µg/mL at thoracic levels at 12, 24, 48, and 72 hours post-injection, respectively (Sung et al., 1978). For WNV treatment, lumbar injection could potentially achieve higher and more sustained drug levels in the CNS. However, data on brain parenchyma concentrations following this route are lacking, warranting further investigation.

**Figure 6:**
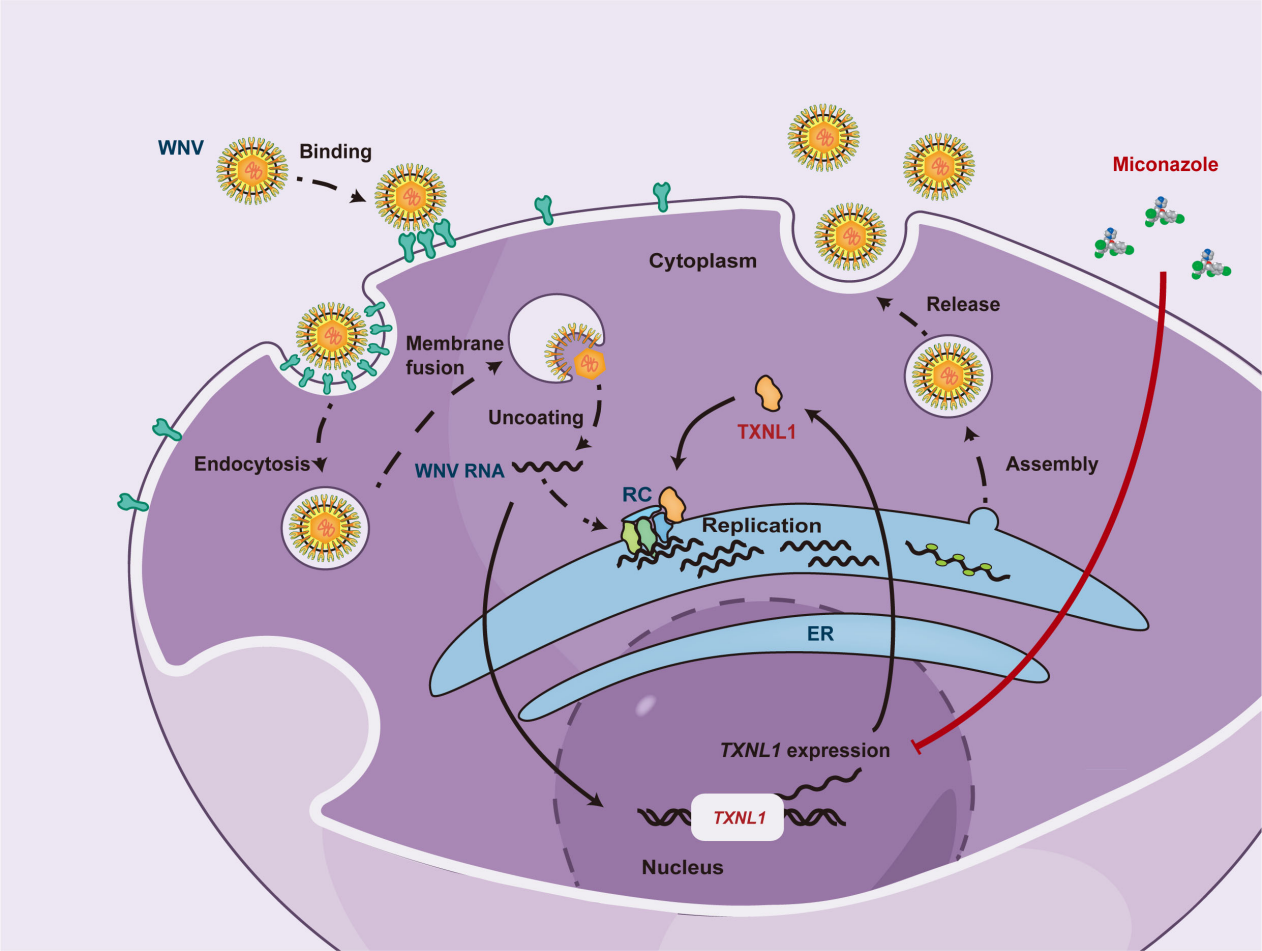
Proposed mechanism of miconazole-mediated inhibition of WNV replication. Miconazole downregulates TXNL1, a host factor required for viral replication.

Thus, we analyzed published transcriptome data on miconazole to identify downregulated host factors. Using siRNA knockdown, overexpression, drug inhibition, and replicon experiments, we confirmed that TNXL1 is likely a contributing factor in miconazole’s ability to inhibit WNV replication. TXNL1, widely expressed and part of the thioredoxin family of redox-active proteins, plays a critical role in regulating the cell cycle, promoting apoptosis, reducing oxidative phosphatase, and enhancing oxidative stress resistance. A study on autoimmune diseases demonstrates that miconazole significantly inhibits the cGAS-STING-IFN pathway (Luo et al., 2025). However, this pathway may not be involved in miconazole action, as it is a classical antiviral pathway. Conversely, TXNL1 has been reported to interact with the para-myxovirus V protein to promote viral replication (Wang C. et al., 2018), aligning with our findings. However, how TXNL1 affects virus replication remains unknown. Research shows that TXNL1 is a stoichiometric component of the 19S regulatory particle of the 26S proteasome and interacts with Rpn11, a subunit of the 19S (Andersen et al., 2009). In tombusvirus studies, Rpn11p interacts with viral replication proteins, acting as a “matchmaker” that recruits viral and host proteins to the viral RC (Prasanth et al., 2015). Thus, during flavivirus infection, we hypothesize that intracellularly expressed TXNL1 may serve as a key regulator of the assembly and function of the viral RC, participating in viral replication (**Figure 6**). In contrast, miconazole inhibits TXNL1 expression, impairing RC function and thereby suppressing viral replication and infection.

While this study demonstrates that miconazole inhibits WNV replication by downregulating TXNL1, the precise downstream mechanism by which TXNL1 facilitates viral replication requires further investigation. Research has shown that the replication of various RNA viruses is highly sensitive to the host cell’s redox state. Moderately elevated reactive oxygen species (ROS) level can enhance the formation of viral replication complexes and improve RNA synthesis efficiency (Gullberg et al., 2015). Consequently, miconazole-induced downregulation of TXNL1 may reduce the level of ROS in cell, thereby inhibiting viral replication. In future experiments, we plan to examine changes in ROS levels regulated by miconazole or TXNL1. Besides, TXNL1, a component of the 19S regulatory particle of the 26S proteasome, interacts with the deubiquitinase Rpn11 (Andor et al., 2023). During viral infections, the proteasome serves a dual purpose: it can degrade viral proteins to limit infection, but some viruses exploit proteasome components to enhance their replication. Rpn11 has been shown to function as a “matching molecule,” recruiting viral replication proteins and host factors to the replication complex (Molho et al., 2021). Therefore, downregulation of TXNL1 may disrupt this protein interaction network, thereby inhibiting viral RNA replication and this should be confirmed in the future study.

This study has several limitations. First, PX-12, the broad-spectrum thioredoxin inhibitor used, primarily targets Trx1, which may lead to non-specific oxidative stress effects. Consequently, its pharmacological results serve only as supplementary evidence, with the core mechanistic conclusions relying mainly on genetic manipulations such as siRNA knockdown and overexpression. Second, further research is needed to determine whether miconazole affects intracellular reactive oxygen species levels and contributes to the antiviral effect by regulating TXNL1. Additionally, the pharmacokinetic characteristics and potential toxicity in the central nervous system require systematic evaluation to assess its clinical translation potential.

## 4 Materials and methods

### 4.1 Cell culture and viruses

SH-SY5Y (ATCC, Manassas, VA) and Huh7 cells (Chinese Academy of Sciences, Shanghai) were cultured in DMEM at 37°C with 5% CO_2_. Compounds from Selleck Chemicals (Houston, TX) were dissolved in dimethyl sulfoxide (DMSO) (10 mM) and stored at –80°C. All compounds were dissolved in DMSO and added to cell culture medium at a final concentration ≤ 0.1%.YFV strain KY587416 was isolated from an imported Chinese case; JEV SA14 (U14163) was provided by the China CDC. Infectious clones of TBEV Zmeinogorsk-5 (KY069125) and WNV NY2000 (AF404756) were synthesized in-house. YFV, TBEV, and WNV work was done under BSL-3; JEV under BSL-2.

### 4.2 High-throughput screening for anti-WNV compounds

A high-throughput screening assay was performed to identify small molecules with anti-WNV and anti-JEV activity. In the high-throughput antiviral screening assay, the Z’ factor was > 0.5 and the signal-to-noise ratio (SNR) was > 10. SH-SY5Y cells were plated at 1 × 10^4^ cells per well in 96-well plates. After 24 hours, cultures were infected with WNV (MOI = 0.3) or JEV (MOI = 0.3) in 100 µL DMEM containing 2% FBS and 10 µM of each compound. Following a 4 hours incubation, infected cells were quantified by immunofluorescence with immunofluorescence assay (see below). The inhibition rate was calculated as: Inhibition Rate (%) = [1 − (infectivit_Compound_/infectivity_DMSO_)] × 100%. Drugs that exhibiting an inhibition efficiency of > 50% against both viruses and no prior confirmed antiviral activity were designated for a second-round validation. Brief descriptions of those candidates is provided in **Supplementary Table 1**.

### 4.3 Immunofluorescence assay

Assays were performed following established protocols. Cells were fixed with methanol at -20°C for 25 min, then blocked with 3% BSA for 2 h at room temperature. Subsequently, cells were treated with primary antibodies against the virus overnight at 4°C. After two times of washes, cells were stained for 2 h at room temperature with AF-488-conjugated donkey anti-rabbit IgG (Thermo Fisher Scientific, San Diego, CA, USA) and then for 10 min with DAPI (Sigma–Aldrich, St. Louis, MO, USA) (nuclei labeling). Imaging was done using a Cytation 5 system. The number of infected cells (fluorescence intensity > 5000) and total cells were quantified with Gen5 3.10 software, and infectivity was normalized to the control group using GraphPad Prism 10.1.2(GraphPad Software, CA, USA).

### 4.4 Determination of EC_50_, CC_50_ and SI

The half-maximal effective concentration (EC_50_) and half-maximal cytotoxic con-centration (CC_50_) values were determined by dose–response analysis. SH-SY5Y cells were infected with WNV and treated with compound at 0.625–40 µM. EC_50_ is the concentration that reduces infection by 50% relative to the DMSO control; CC_50_ is the concentration that reduces cell viability by 50%. Both EC_50_ and CC_50_ values were determined using GraphPad Prism software (version 10.1.2). The selectivity index (SI) was calculated as the ratio of CC_50_ to EC_50_ (SI = CC_50_/EC_50_).

### 4.5 RT-PCR

Huh7 cells, SH-SY5Y cells, and mouse tissues were lysed with TRIzol (TaKaRa, Shiga, Japan) to extract total RNA. This RNA was then reverse transcribed using the PrimeScript RT Master Mix kit (TaKaRa) and amplified by PCR with SYBR Premix Ex Taq (TaKaRa) using respective primer pairs (**Supplementary Table 2**) on an ABI 7300 system (Applied Biosystems, Carlsbad, CA, USA). Expression levels of WNV RNA, candidate host genes, and the internal reference gene glyceraldehyde-3-phosphate dehydrogenase (GAPDH) were determined by normalizing against GAPDH and the no-drug-treatment infection group using comparative cycle threshold values (2−ΔΔCt). The relative quantity (RQ) of target genes was calculated accordingly.

### 4.6 siRNA and OE plasmid transfection

SH-SY5Y cells were seeded in 24-well plates to reach 80% confluency. For each transfection, 2 µL of Lipo2000 transfection reagent (Invitrogen, San Diego, CA, USA) reagent and 2 µL of siRNA or overexpression (OE) plasmid (final 40 nM) were diluted separately in 50 µL of serum-free medium. After 5 minutes, the two dilutions were mixed together, incubated for 20 minutes at room temperature, and added to the cells. Six hours post-transfection, the medium was replaced with complete medium for another 48 hours of incubation.

### 4.7 Virus binding assay

Virus binding and internalization assay were conducted as described before[18]. Briefly, SH-SY5Y cells were seeded into collagen-coated 24-well plates. When reached 100% confluency, the cells were inoculated with WNV (MOI = 10), together with either 20 μM miconazole or an equivalent volume of DMSO. After incubated at 4 °C for 2 hours to allow virus binding, the supernatant was removed, and the cells were washed twice with ice-cold PBS to remove unbound virus. Finally, Cells were lysed with TRIzol re-agent, total RNA was extracted, and subjected to RT qPCR to quantify the amount of WNV RNA bound to the cell surface.

### 4.8 Virus internalization assay

SH-SY5Y cells were seeded into collagen-coated 24-well plates. When the cells reached 100% confluency, the culture medium was aspirated. The cells were then incubated with WNV (MOI = 10), together with either 20 μM miconazole or an equal volume of DMSO, at 4 °C for 2 hours to permit viral binding. Following incubation, the medium was removed, and the cells were washed once with ice-cold PBS. Subsequently, 500 μL of fresh pre-warmed medium was added, and the plates were transferred to a 37 °C for 1.5 hours to allow for viral internalization. Then, the medium was discarded, and the cells were treated with a Proteinase K working solution (1 mg/mL) at 4 °C for 30 minutes to remove any remaining virions attached to the cell surface. The cells were then collected and washed twice with PBS. Total RNA was extracted and subjected to RT qPCR to quantify the levels of internalized WNV RNA.

### 4.9 Viral membrane fusion kinetic assay

WNV was first labeled by incubating with the lipophilic fluorescent dye DiD (Thermo Fisher Scientific, San Diego, CA, USA) at a concentration of 10 µM for 1 hour at room temperature. Unincorporated dye was removed via ultracentrifugation at 4 °C and 28,000 rpm for 4 hours. SH-SY5Y cells were seeded into collagen-coated 24-well plates. Upon reaching 100% confluency, the cells were infected with labeled virus (WNV^DiD^, MOI = 1), concurrently with the addition of either DMSO (vehicle control), 20 µM miconazole, or 25 mM NH_4_Cl (positive control). Another two control groups, consisting of DiD dye alone or unlabeled WNV, were included. The real-time kinetics of vi-ralhost membrane fusion were monitored by measuring DiD fluorescence dequenching using a fluorescence microplate reader. The parameters were as follows: excitation wavelength, 644 nm; emission wavelength, 663 nm; assay temperature, 37 °C; total duration, 8 hours; with data points recorded every 15 minutes.

### 4.10 Viral genome RNA extraction

Viral RNA was extracted from WNV stock solution using the RNAeasy™ Viral RNA Extraction Kit (Beyotime Biotechnology, Suzhou, China) according to the manufacturer’s instructions. Briefly, the viral lysate was prepared by adding lysis buffer to the original viral suspension at a 1:3 ratio. After thorough mixing, an equal volume of binding buffer was added to the mixture. The solution was gently inverted 3–5 times and then transferred entirely to a purification column. The column was subjected to centrifugation at 12,000 × g for 30 seconds, and the flow-through in the collection tube was discarded. Subsequently, 600 µL of Wash Buffer I was added to the column, followed by centrifugation at 12,000 × g for 30 seconds, and the flow-through was again discarded. This was followed by two consecutive washes with 600 µL of Wash Buffer II under the same centrifugation conditions. To remove any residual liquid, the column was centrifuged at 14,000 × g for 2 minutes. Finally, the purification column was placed into the supplied elution tube. Thirty microliters of Elution Buffer were added directly onto the column membrane, incubated at room temperature for 2–3 minutes, and then centrifuged at maximum speed for 30 seconds. The eluate constituted the purified viral genome RNA.

### 4.11 Replication assay

Replicon plasmids carrying the NanoLuc Luciferase reporter gene, based on West Nile virus (WNV) strain, were constructed as we described before. These plasmids were linearized using restriction endonucleases to serve as templates for *in vitro* transcription of replicon RNA, which was performed using the HiScribe T7 ARCA mRNA Kit (New England Biolabs, Ipswich, MA, USA). Subsequently, the transcribed RNA was transfected into SH-SY5Y cells using Lipofectamine 2000 reagent. After six hours of transfection, the supernatant was replaced with fresh medium containing either 20 µM miconazole or DMSO (vehicle control). At 48 hours post-transfection (hpt), cells were fixed and stained with WNV NS1 antibody and DAPI, and visualized using a Cytation 5 imaging system, and the mean fluorescence intensity was quantified with Gen5 3.10 software and normalized to the DMSO-treated control. For NanoLuc detection, the medium was aspirated, and a 10% Nano-Glo substrate reagent was added. Luminescence was then measured using a chemiluminescence imaging system (Clinx Science Instruments, Shanghai, China).

In parallel, WNV genome RNA was transfected into SH-SY5Y cells using Lipofectamine 2000. At 6 hpt, the supernatant was replaced with fresh medium containing either 20 µM miconazole or DMSO, and the cells were further incubated for 3, 6, and 9 hours (corresponding to 9, 12, and 15 hpt, respectively). Total RNA in cells and supernatant was extracted with TRIzol and TRIzol LS reagent. WNV RNA levels were analyzed by RT–qPCR. The replication level of WNV was expressed as the relative quantity (RQ) of WNV RNA in cells compared to the DMSO-treated control group.

### 4.12 Animal experiment

Six-week-old female C57BL/6 mice were purchased from Shanghai Jihui Laboratory Animal Care Co., Ltd. and housed in a specific pathogen-free (SPF) facility with a 12 h light/dark cycle, and free access to food and water. Mice were randomly assigned into three groups, mock group (PBS inoculation with vehicle treatment), model group (WNV inoculation with vehicle treatment), and miconazole group (WNV inoculation with miconazole treatment), using a random number generator, which ensured the homogeneity of initial body weight, gender, and age among the three groups and effectively excluded the interference of individual differences on experimental results. A single-blind design was adopted: all animal manipulations, including viral infection, drug administration, sample collection, body weight measurement, survival monitoring, and subsequent virological and pathological analyses, were performed by researchers who were unaware of the group assignments to avoid subjective bias in the experimental process and data analysis. The sample size was estimated using the log-rank test based on the pre-experimental results, which showed a 20% difference in survival rate between groups; with a two-sided α = 0.05 and a test power of 80% (power = 0.8), the calculated sample size was 10 mice per group for survival monitoring to ensure sufficient statistical power. In addition, 3 mice per group were set for virological and pathological analyses on day 6 post-infection.

Mice were inoculated intracranially with 100 plaque-forming units (PFU) of WNV or an equivalent volume of PBS (mock group). Two hours post-infection, the miconazole group received miconazole (50 mg/kg/day, suspended in corn oil) via oral gavage once daily for 5 consecutive days. The model group received the same volume of DMSO diluted in corn oil by oral gavage, while the mock group received neither viral inoculation nor drug treatment. Body weight was measured daily, and survival status was monitored for 14 days post-infection. Mice that lost ≥20% of their initial body weight or exhibited severe neurological symptoms were euthanized humanely in accordance with animal welfare guidelines. On day 6 post-infection, 3 mice per group were euthanized via isoflurane overdose. Brains were rapidly harvested and divided into two halves: one half was homogenized in TRIzol reagent for total RNA extraction, followed by RT-qPCR to determine the viral load; the other half was fixed in 3.5% paraformaldehyde for 24 hours, embedded in paraffin, and sectioned at 5 μm for viral NS1 immunofluorescence staining. Stained sections were scanned using a digital pathological section scanner (LG-S80, Servicebio Inc., China), and quantitative analysis was performed using ImageJ. All experimental procedures complied with the ARRIVE guidelines and were conducted in strict accordance with the approved animal care and use protocols. All animal experiments were approved by the Laboratory Animal Ethics Committee at the Naval Medical University, Shanghai, China.

### 4.13 Statistic analysis

Data are presented as arithmetic mean ± standard deviation (SD) from at least three biologically independent experiments. Statistical analyses were performed using GraphPad Prism 10.1.2 (GraphPad Software, USA). For comparisons among multiple groups, one-way analysis of variance (ANOVA) was applied, followed by Bonferroni *post-hoc* test for pairwise comparisons. Statistical significance was defined as P < 0.05. Specific P values are indicated in the figure legends or reported as *P < 0.05, **P < 0.01, ***P < 0.001, and ****P < 0.0001. No data were excluded from the analyses. The exact number of independent experiments (n) and biological replicates is specified in each figure legend. The survival analysis for the *in vivo* efficacy experiment in mice was performed using the Kaplan-Meier method, and statistical differences between the miconazole-treated group and the control group were evaluated using the log-rank test.

## Data Availability

The raw data supporting the conclusions of this article will be made available by the authors, without undue reservation.
