# Supplementary material for: Downregulation of thioredoxin-like 1 contributes to miconazole’s antiviral activity against West Nile virus
Source: Front Cell Infect Microbiol. 2026 Jul 21;16:1789670. doi: 10.3389/fcimb.2026.1789670 (PMC13433433; doi:10.3389/fcimb.2026.1789670)
Supplement: Supplementary file 1 [file Table1.docx]

**Miconazole inhibits West Nile virus replication through downregulating Thioredoxin Like 1**

**Liancheng Xia^1,2,†^, Zhenghan Luo^1,2,3,†^, Yanhua He^1,2,†^, Haoran Peng^1,2^, Xu Zheng^1,2^, Xijing Qian^1,2^, Cuiling Ding^1,2^, Ping Zhao^1,2,*^, Yangang Liu^1,2,*^, Zhongtian Qi^1,2,*^**

^1^Department of Microbiology, Faculty of Naval Medicine, Naval Medical University, Shanghai 200433, China;

^2^Key Laboratory of Biological Defense, Ministry of Education, Naval Medical University, Shanghai 200433, China;

^3^Center for Disease Prevention and Control of Eastern Theater of Chinese PLA, Nanjing, Jiangsu 210002, China;

***Correspondence:**

qizt@smmu.edu.cn (Z.Q.); lyg@smmu.edu.cn (Y.L.); pnzhao@163.com (P.Z.)

**†**These authors contribute equally to this work.

**Table S1. Information of the candidate small molecules**

| **Name** | **Cat** | **CAS Number** | **M.w.** | **Formula** | **Information** |
| --- | --- | --- | --- | --- | --- |
| **Miconazole nitrate** | HY-B0454A | [22832-87-7](https://www.medchemexpress.cn/cas/22832-87-7.html) | 479.14 | C_18_H_15_Cl_4_N_3_O_4_ | Miconazole nitrate (R18134 nitrate) is an imidazole antifungal agent It works by inhibiting the synthesis of ergosterol, an important component of fungal cell membranes. |
| **Neflamapimod** | HY-10328 | [209410-46-8](https://www.medchemexpress.cn/cas/209410-46-8.html) | 436.26 | C_19_H_9_Cl_2_F_2_N_3_OS | Neflamapimod (VX-745) is an effective, blood-brain barrier-penetrating, highly selective p38α inhibitor with an IC50 value of 10 nM for p38α and 220 nM for p38β. Neflamapimod (VX-745) exhibits anti-inflammatory activity. |
| **Vilazodone Hydrochloride** | HY-14261 | [163521-08-2](https://www.medchemexpress.cn/cas/163521-08-2.html) | 477.99 | C_26_H_28_ClN_5_O_2_ | Vilazodone hydrochloride (EMD 68843 Hydrochloride) is an inhibitor of the serotonin reuptake transporter (SER) and a partial agonist of the 5-HT1A receptor. |
| **Dacomitinib** | HY-13272 | [1110813-31-4](https://www.medchemexpress.cn/cas/1110813-31-4.html) | 469.94 | C_24_H_25_ClFN_5_O_2_ | Dacomitinib (PF-00299804) is a specific, irreversible inhibitor of the ERBB family, with IC₅₀ values of 6 nM, 45.7 nM and 73.7 nM for EGFR, ERBB2 and ERBB4 respectively. |
| **Cisatracurium besylate** | HY-13596 | [96946-42-8](https://www.medchemexpress.cn/cas/96946-42-8.html) | 1243.48 | C_65_H_82_N_2_O_18_S_2_ | Cisatracurium besylate (51W89) is a non-depolarising neuromuscular blocking agent that antagonises the effects of acetylcholine by inhibiting neuromuscular transmission. |
| **Mirtazapine** | HY-13596 | [96946-42-8](https://www.medchemexpress.cn/cas/96946-42-8.html) | 1243.48 | C_65_H_82_N_2_O_18_S_2_ | Cisatracurium besylate is a neuromuscular blocking agent or skeletal muscle relaxant belonging to the non-depolarising neuromuscular blocking agent class. It is used as an adjunct in anaesthesia to facilitate tracheal intubation and to relax skeletal muscles during surgery or mechanical ventilation. |
| **Rimonabant** | HY-14136 | [168273-06-1](https://www.medchemexpress.cn/cas/168273-06-1.html) | 463.79 | C_22_H_21_Cl_3_N_4_O | Rimonabant (SR141716) is a potent, selective, blood-brain barrier-permeable antagonist of the central cannabinoid receptor 1 (CB1), with a Ki value of 1.8 nM. Rimonabant (SR141716) also inhibits mycobacterial membrane protein 3 (MMPL3). |
| **Lvacaftor** | HY-13017 | [873054-44-5](https://www.medchemexpress.cn/cas/873054-44-5.html) | 392.49 | C_24_H_28_N_2_O_3_ | Ivacaftor (VX-770) is an effective, orally administered CFTR potentiator with EC50 values of 100 nM for G551D-CFTR and 25 nM for F508del-CFTR. |
| **Itopride hydrochloride** | HY-B0732 | [122892-31-3](https://www.medchemexpress.cn/cas/122892-31-3.html) | 394.89 | C_20_H_27_ClN_2_O_4_ | Itopride (HSR803 free base) is an effective dopamine 2 (dopamine-2) antagonist and acetylcholinesterase (AChE) inhibitor. Itopride enhances gastric motility through its anti-dopaminergic and anti-acetylcholinesterase actions, and may be used as a gastrointestinal prokinetic agent. |
| **Vinorelbine ditartrate** | HY-12053A | [125317-39-7](https://www.medchemexpress.cn/cas/125317-39-7.html) | 1079.11 | C_53_H_66_N_4_O_20_ | Vinorelbine ditartrate is an antimitotic agent capable of inhibiting the proliferation of HeLa cells, with an IC₅₀ value of 1.25 nM. |

**Table S2. Sequences of primer pairs specific to target gene**

| **Target Gene** | **Sequences of qRT-PCR Primer Pair (5’-3’)** |
| --- | --- |
| **GAPDH** | Forward primer: TGGGCTACACTGAGCACCAG  Reverse primer: AAGTGGTCGTTGAGGGCAAT |
| **WNV** | Forward primer: GAACCACCATATAGAACCTG  Reverse primer: TTCCTCTCGAGAGCACATTCT |
| **SELT** | Forward primer: GGTACCCAGACATCCGCATT  Reverse primer: CAGTGCTGATAGGTGGTGTCT |
| **TXNL1** | Forward primer: AGAGTCCCGGGAGAAGACTC  Reverse primer: TGCCAACTACTCGTTTGAAGTC |
| **ANXA1** | Forward primer: TGCAAGAAGGTAGAGATAAAGACAC  Reverse primer: TCAGTGTTTCATCCAGGGGC |
| **CASP6** | Forward primer: CACCCGGCAGTGTCAACT  Reverse primer: GCATTGAGGCAAAACAGGGA |
| **TXNRD1** | Forward primer: GAAAGGCAAGAACGGCGATG  Reverse primer: GAGAGTCTTGCAGGGCTTGT |
| **PRDX3** | Forward primer: GTCTTGCACTAAGATCAAGCCA  Reverse primer: AAACTAGCTAGCCAGCCACC |
| **DNAJC10** | Forward primer: GCTTAGTGGCATGTTGTTTCTCA  Reverse primer: CGTTGAAGGTGGTGGGTGTA |
| **APEX1** | Forward primer: GCAGATACGGGGTTGCTCTT  Reverse primer: ATTTTTACCGCGTTGCTCGC |
| **CAT** | Forward primer: AGTGATCGGGGGATTCCAGA  Reverse primer: CCACCCTGATTGTCCTGCAT |
| **FOSL1** | Forward primer: CTGGTGCCAAGCATCAACAC  Reverse primer: TCTTCCTCCGGGCTGTTCA |
